# AGE- AND ETHNICITY-RELATED DISPARITIES IN TECHNOLOGY USE AMONG HIGH-RISK VETERANS

**DOI:** 10.1093/geroni/igz038.1213

**Published:** 2019-11-08

**Authors:** Kiranmayee Muralidhar, Willy Marcos Valencia, Kaicheng Wang, Diana Ruiz, Carlos Gomez-Orozco, Stuti Dang

**Affiliations:** 1 Department of Public Health Sciences, University of Miami Miller School of Medicine, Miami, Florida, United States; 2 Miami Veterans Affairs Healthcare System- GRECC, Miami, Florida, United States; 3 South Florida Veterans Affairs Foundation for Research and Education Miami, Miami, Florida, United States

## Abstract

Using predictive analytic modelling, the Veterans Affairs has identified Veterans considered to be High Need High Risk (HNHR) requiring increased support. This pilot study sent needs assessment questionnaires to 1112 HNHR Veterans to better understand gaps regarding technology use, access, physical function, and mobility. There were 341(30.7%) respondents: 270(80.4%) Non-Hispanic, 64(18.8%) Hispanic/Latino; 210(61.6%) White, 119(34.9%) Black/African Americans; and 310(90.4%) had ≥high school education. Average Barthel(ADL) score was 81.5±22.8 and Lawton(IADL) score was 5.8±2.2. Younger Veterans (age<70) were more likely able to use Internet ((117(65%) vs 74(46%)),(p≤0.01) and email (106(58.9%) vs 67(41.6%),( p≤0.01). They were also more likely enrolled in MyHealtheVet (87(48.3%) vs 58(36%),(p=0.043). Secure messaging was used by 62(34.3%) younger and 37(23%) older Veterans,(p=0.026). More higher functioning Veterans (140(55.1%)) used email than lower functioning (33(37.9%)),(p=0.018). Among higher functioning Veterans, 148(58.3%) were willing to use videoconference for care coordination and 116(45.7%) owned a smartphone or computer with camera for this; more than lower functioning Veterans (33(37.9%) and 28(32.2%)), (p≤0.01 for both). Less dependent Veterans preferred to be contacted via cellphone (88(62.4%)) or Internet (10(7.1%)) compared to the more dependent (96(48%) and 6(3%)) respectively (p=0.01). Only 71(44.1%) of older Veterans were willing to use videoconference (p≤0.01) and 54(33.5%) owned a smartphone or computer with camera,(p≤0.01). There are significant variations in technology use by age and ethnicity. However, although there are differences by functional ability, a significant number of disabled veterans are willing and able to use technology, and this may provide a way to address access barriers in this population.

